# Different therapeutic responses to osimertinib as first-line treatment for cavitated and non-cavitated squamous cell lung cancer with rare EGFR exon 19 deletion: two case reports and a literature review

**DOI:** 10.3389/fonc.2025.1626665

**Published:** 2025-10-20

**Authors:** Jing Ren, Liang Xiao Cheng, Rui Zhang, Zhi Zou, Yongzhao Zhou, Xi Zheng

**Affiliations:** ^1^ The Integrated Care Management Center, Outpatient Department, West China Hospital, Sichuan University, Chengdu, China; ^2^ Institute of Respiratory Health and Multimorbidity, West China Hospital, Sichuan University, Chengdu, China; ^3^ Department of Pneumoconiosis Pulmonary and Critical Care Medicine, West China School of Public Health and West China Fourth Hospital, Sichuan University, Chengdu, China; ^4^ Department of Respiratory Medicine, Meishan People's Hospital, Sichuan Province, Meishan, China; ^5^ Lung Cancer Center/Lung Cancer Institute, West China Hospital, Sichuan University, Chengdu, China; ^6^ Department of Thoracic Surgery, West China Hospital, Sichuan University, Chengdu, China

**Keywords:** squamous cell lung cancer, cavitation, non-cavitated, epidermal growth factor receptor tyrosine kinase inhibitors, osimertinib

## Abstract

**Background:**

Cavitation is a special radiological feature of lung cancer, commonly observed in squamous cell lung cancer (SqCLC). This study intended to report two cases of cavitated and non-cavitated SqCLCs treated with epidermal growth factor receptor tyrosine kinase inhibitors (EGFR-TKIs) and discuss their different therapeutic responses.

**Case presentation:**

Two SqCLC patients with EGFR exon 19 deletions were treated with osimertinib as first-line therapy. One patient presented with a thick-walled cavitated lesion, while the other had a non-cavitated mass. After 3 months of osimertinib treatment, the cavitated SqCLC case showed disease progression. This patient subsequently underwent surgical resection of the primary tumor, followed by chemotherapy and immunotherapy, achieving an overall survival of 41 months to date. In contrast, the non-cavitated SqCLC case responded better to osimertinib, achieving partial remission with a progression-free survival of 14 months. Upon subsequent growth in both the primary lesion and lymph nodes, this patient began treatment with chemotherapy combined with immunotherapy, with a current overall survival of 31 months.

**Conclusions:**

These cases suggest that osimertinib may be less effective as a first-line treatment for EGFR driver gene positive cavitated SqCLC than non-cavitated cases. Further research is needed to evaluate whether combining EGFR-TKIs with other therapies provides greater benefits than EGFR-TKIs alone for EGFR-positive cavitated SqCLC.

## Background

Cavitated lung cancer accounts for approximately 2%–25% of all lung cancers, with squamous cell lung cancer (SqCLC) being the most common histological type (1, 2). The cavitation is often thought to result from rapid tumor growth, insufficient blood supply, bronchial obstruction, or vascular invasion leading to central necrosis or liquefaction (3). Studies suggest that cavitated SqCLC may be associated with epidermal growth factor receptor (EGFR) overexpression, which could promote tumor growth and angiogenesis (4). Cavitated tumors are typically 1.5 times larger in diameter than non-cavitated tumors, and patients often present with more pronounced symptoms such as hemoptysis, infection, and weight loss (5, 6). While some research indicates that cavitated lung cancer is associated with poor prognosis, other studies suggest no significant difference in progression-free survival (PFS) or overall survival (OS) compared to non-cavitated lung cancer (7).

The mutation rate of EGFR in SqCLC is notably lower than in lung adenocarcinoma (LUAD), and SqCLC generally exhibits a poorer response to EGFR tyrosine kinase inhibitors (TKIs) (8). However, there is limited research on the response of different types of SqCLC to targeted therapies. At present, there is no consensus on whether cavitated SqCLC constitutes a distinct subtype, and comparative studies of TKI treatment for cavitated vs. non-cavitated SqCLC with gene mutations are scarce. Here, we reported two cases of cavitated and non-cavitated SqCLCs treated with EGFR-TKIs and provided a review of the relevant literature.

## Case presentation

### Case one

**Figure 1:**
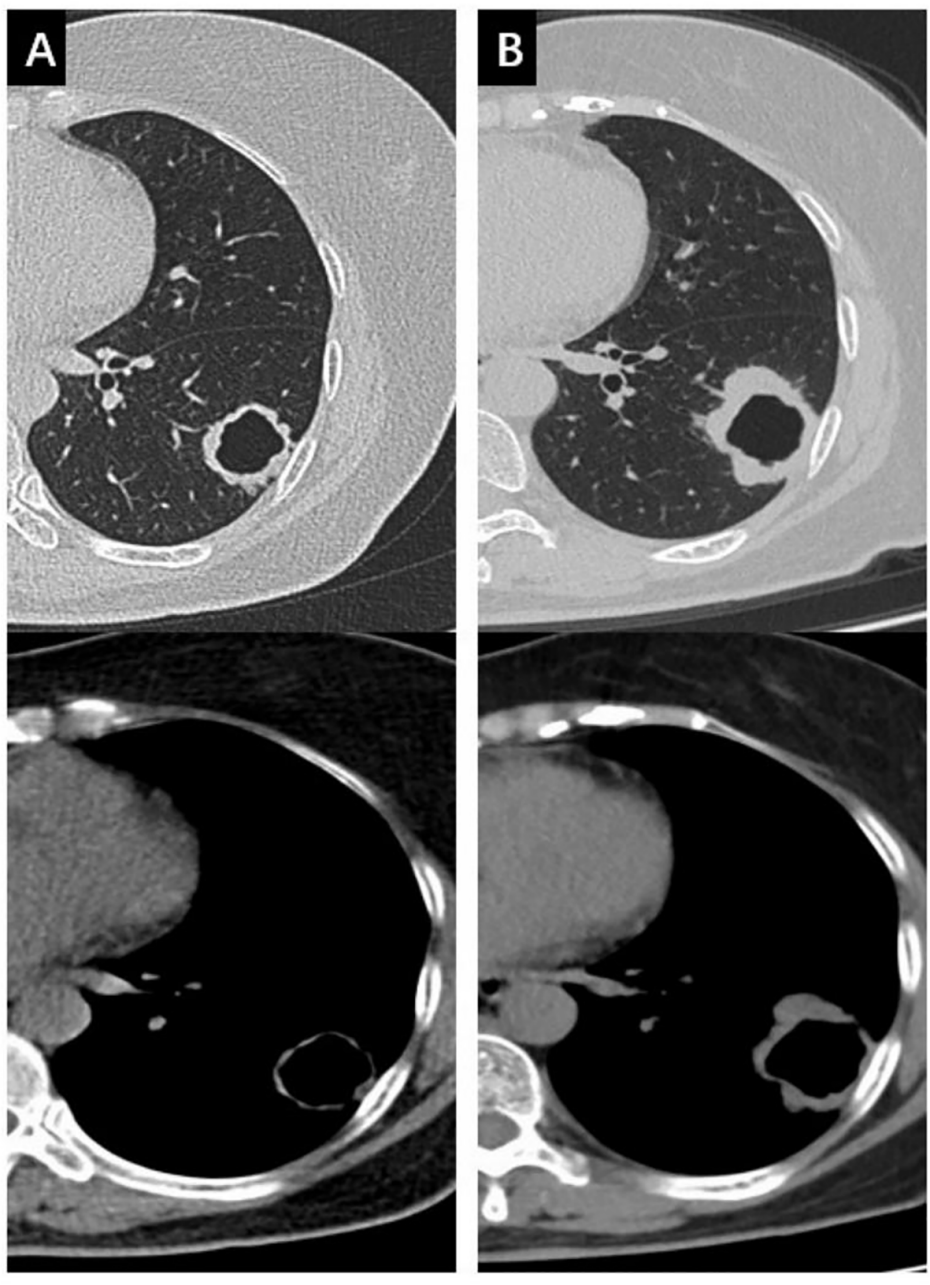
The changes of the mass in the left lower lobe during anti-tuberculosis treatment in both the lung window and the mediastinal window. **(A)** the mass at baseline; **(B)** the mass enlarged 6 months later after anti-tuberculosis treatment.

A 69-year-old female with no smoking or family history of cancer, but a history of pulmonary tuberculosis, presented with a thick-walled cavity (31x29 mm) in the left lower lobe on chest CT. Preliminary diagnosis didn’t rule out the possibility of tuberculosis recurrence, so the patient received six months of anti-tuberculosis treatment at a local hospital. However, follow-up CT showed progression of the primary lesion and enlargement of left hilar lymph nodes, suggesting intrapulmonary metastasis (**Figure 1**). The cavitated mass was subsequently confirmed as SqCLC by CT-guided percutaneous lung biopsy, staged as cT2aN1M1a (IVA, AJCC 8th edition). Genetic testing showed an EGFR exon 19 deletion and PTEN mutation, with a PD-L1 negative expression by immunohistochemistry.

**Figure 2:**
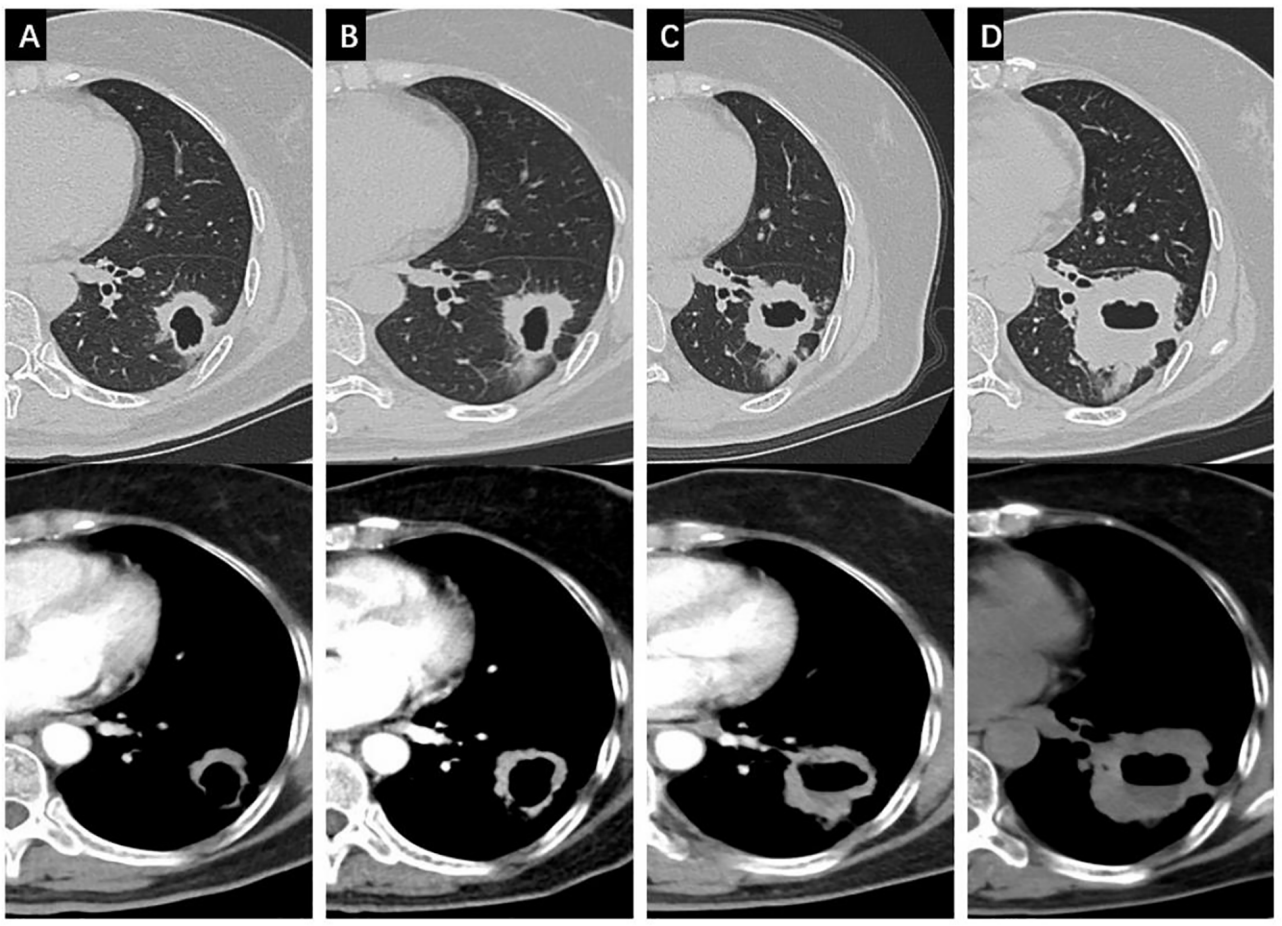
Changes of the cavitated SqCLC in the left lower lobe in both the lung window and the mediastinal window. **(A)** progression of the primary lesion after 3 months of osimertinib; **(B)** progression of the primary lesion after 6 months of osimertinib; **(C)** progression of the primary lesion after 12 months of Osimertinib; **(D)** progression of the primary lesion after 15 months of osimertinib.

Osimertinib was initiated as first-line therapy, but after 3 months, the primary lesion slightly enlarged. Because the patient rejected radiotherapy and chemotherapy, continued treatment with osimertinib lasted for 15 months, and therapeutic evaluation was disease progression (**Figure 2**). Since the solitary solid nodule in the right lower lobe remained unchanged during treatment and is unlikely to be metastatic, the disease was staged as cT2aN1M0 (IIB). Additionally, due to the lesion’s proximity to major pulmonary blood vessels and the high risk of fatal complications such as massive hemoptysis and infection in cavitated SqCLC, resection of the cavitated lesion was recommended (9). Postoperative pathology confirmed SqCLC without adenocarcinoma components. The pathological stage was pT2bN2M0 (IIIA). Genetic testing also showed EGFR exon 19 deletion (2.13%) and PTEN mutation (14.74%). Post-surgery, she received four cycles of chemotherapy (Taxol + Platinum, TP) with immune checkpoint inhibitor (Endostar) therapy. No recurrence or metastasis has been observed on follow-up, achieving an OS of 41 months to date.

### Case two

A 68-year-old male smoker (27 pack-years) presented with a solid mass (39 x 32 mm) in the right middle lobe, along with partial enlargement of right hilar and mediastinal lymph nodes on chest CT. PET/CT revealed increased metabolic activity in the mass, suggesting possible metastasis to the pleura and mediastinal lymph nodes. The mass in the right middle lobe was confirmed SqCLC by CT-guided percutaneous lung biopsy, staged as cT2aN2M1a (IVA). Genetic testing revealed EGFR exon 19 deletion and a PIK3CA mutation, with a positive PD-L1 expression (TPS = 20%) by immunohistochemistry.

**Figure 3:**
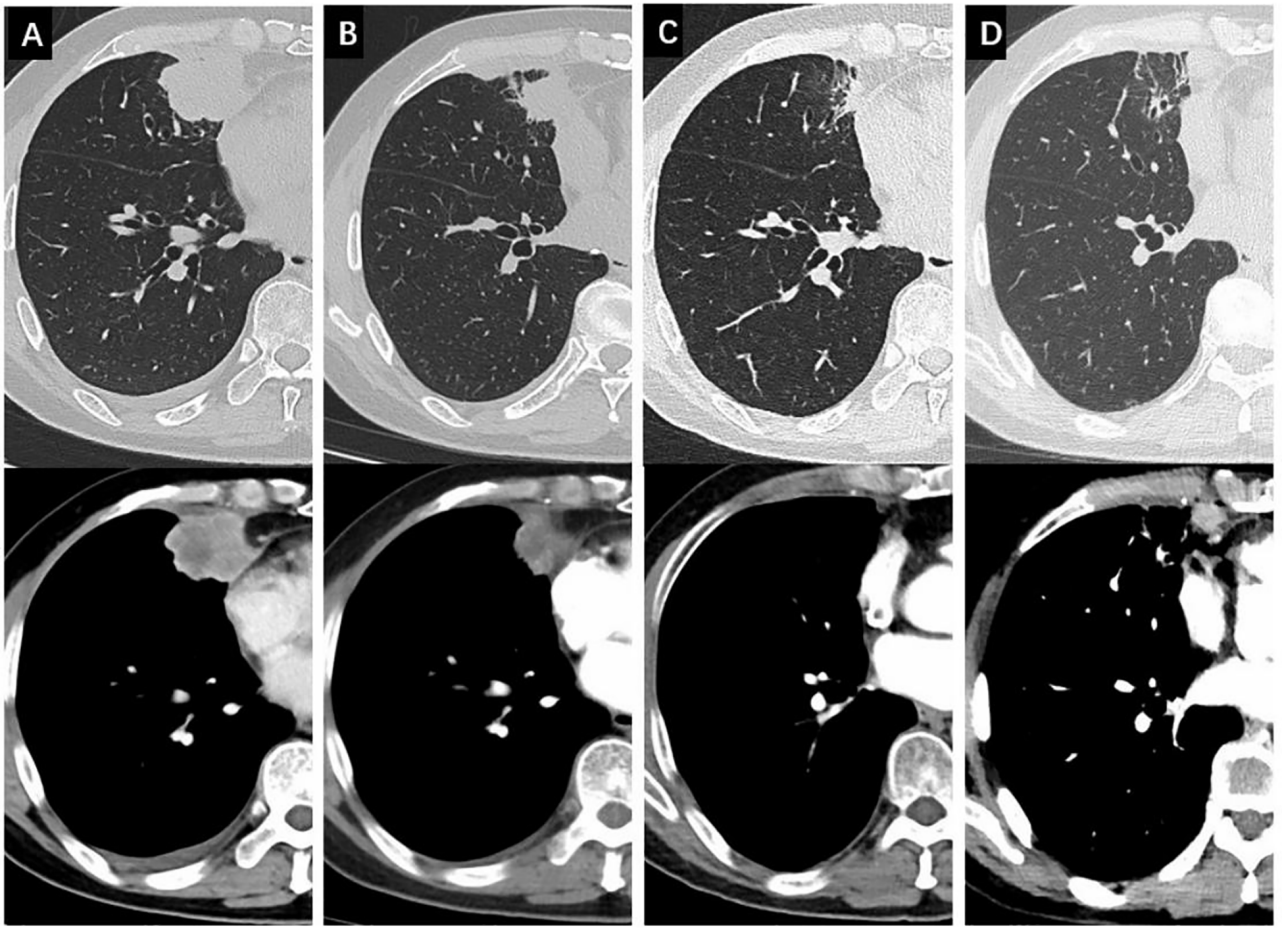
Changes of the non-cavitated SqCLC in the right middle lobe in both the lung window and the mediastinal window. **(A)** baseline CT before osimertinib treatment; **(B)** changes of the primary lesion after 1 months of osimertinib; **(C)** changes of the primary lesion after 14 months of osimertinib; **(D)** changes of the primary lesion after 16 months of osimertinib with a newly emerged lesion in the right diaphragmatic angle.

Osimertinib was used as the first-line therapy. One month later, follow-up CT showed remission of both the primary lesion (29 x 20 mm) and lymph node, with continued shrinkage for over 14 months (18 x 12 mm). However, after 16 months, resistance to osimertinib developed, and follow-up CT showed a new metastatic lesion at the right diaphragmatic angle (**Figure 3**) as well as in mediastinal lymph nodes. The disease was staged as cT1bN2M1a (IVA). Six cycles of chemotherapy (Taxol + Platinum, TP) combined with immune checkpoint inhibitor (Tislelizumab) therapy were applied, and the disease maintained stable with an OS of 31 months to date.

## Discussion

SqCLC is the second most common lung cancer subtype, accounting for 25%-30% of cases (10). Osimertinib, initially approved for T790M-positive non-small cell lung cancer, has shown better efficacy in LUAD with EGFR mutations when used as first-line therapy (11). To our knowledge, this is the first reported case of cavitated SqCLC with an EGFR exon 19 deletion treated with osimertinib as first-line therapy.

Cavitation appears on chest CT as a central, gas-filled, low-attenuation area within a mass, surrounded by cavity walls of variable thickness. In lung cancer, cavities are typically thick-walled (>4 mm) and are more frequently observed in SqCLCs. Liu et al. reported that cavitated lung cancers are associated with a poorer prognosis compared with non-cavitated tumors (12).

A literature review identified only two reported cases of EGFR exon 19 deletion in SqCLC treated with osimertinib as a first-line therapy (**Table 1**, first two studies) (13, 14). Nishimura T et al. reported one patient passed away after three weeks with disease progression, but details remain uncertain. Despite a non-cavitated SqCLC, CT scans showed central liquefactive necrosis. Moreover, osimertinib treatment lasted only 18 days, and the EGFR exon 19 deletion detected in the initial small biopsy was absent in autopsy specimens. In contrast, Peng M et al. reported one patient with both EGFR exon 19 deletion and T790M mutation demonstrating a remarkable response. After three months of osimertinib, CT scans revealed significant reduction in lung lesions and lymph nodes, and surgical resection confirmed a pathological complete response. This case suggests that osimertinib may be effective in SqCLC with “primary” resistance mechanisms (e.g., EGFR T790M mutation). Additionally, other studies (**Table 1**, cases 3 and 4) have reported partial remission in EGFR exon 19 deletion SqCLC patients after disease progression with radiotherapy and chemotherapy, further supporting osimertinib’s potential efficacy in these cases (15, 16).

**Table 1:** Case reports of osimertinib therapy for patients with EGFR 19 exon del SqCLC.

Case	Author	Age	Sex	Smoking history	Clinical stage	Cavitated SqCLC	EGFR mutation	Osimertinib as first-line therapy	Response
1	Nishimura et al. (12)	83	M	Yes	IVA	Non-cavitated with central liquefaction	19del	Yes	PD *
2	Peng et al. (13)	50	M	Yes	IIIB	Non-cavitated	19del/T790M	Yes	CR
3	Shoji et al. (14)	67	F	Yes	IIIB	Non-cavitated	19del	No	PR
4	Maeda et al. (15)	57	M	No	IIIC	Non-cavitated	19del/T790M	No	PR
5	Current study case one	69	F	No	IIIA	cavitated	19del	Yes	PD
6	Current study case two	68	M	Yes	IVA	Non-cavitated	19del	Yes	PR

SqCLC, squamous cell lung cancer; M, male; F, female; PD, progressive disease; CR, complete remission; PR, partial response.

*In this case, EGFR 19del was detected in a small biopsy but not confirmed in the autopsy specimens. Chest CT revealed central liquefactive necrosis, a characteristic change preceding cavity formation. Additionally, the patient received osimertinib treatment for only 18 days.

In this study, we reported two cases with similar diagnoses and treatments but different outcomes. One factor influencing EGFR-TKI efficacy may be the adjusted variant allele frequency (aVAF) and the presence of coexisting genetic mutations. Balazs et al. found that EGFR-TKI treatment significantly prolonged PFS and OS in 70% of patients with high EGFR-aVAF (17). In case one, the postoperative EGFR-aVAF was only 3.55%, and the presence of PTEN mutations, known to contribute to drug resistance, may also explain the poor response to EGFR-TKI therapy (18, 19). PTEN mutations are found in about 15% of SqCLC cases, significantly higher than in LUAD (20). PTEN mutation can lead to sustained activation of negative feedback regulation, which may help predict resistance to EGFR-TKIs (21). Thus, chemotherapy alone or in combination with immunotherapy may be more effective when such resistance to EGFR-TKIs develops (13). Besides, PIK3CA mutations occur in various cancers, at frequencies of 5% to 8% in NSCLC cases and approximately 6.33% in Chinese pan-cancer samples. PIK3CA mutations can promote cellular survival and proliferation, which contribute to resistance to EGFR-TKIs in lung cancer (22). In case two, genetic testing revealed EGFR exon 19 deletion and a PIK3CA mutation, but it still demonstrates good therapeutic effects, which worth further exploration.

Additionally, cavitation is more common in SqCLC and is associated with more aggressive behavior and poorer outcomes compared to non-cavitated SqCLC (5, 6). Hypotheses for cavity formation include poor blood supply, which affects drug delivery, and tumor cells shedding into the airways, causing local obstruction and cavity formation. The complex cavity wall composition may also create a barrier to drugs, contributing to TKI resistance. Besides, cavitated SqCLC is often misdiagnosed as an infectious disease (6, 23). For example, in case one, preliminary diagnosis didn’t rule out tuberculosis recurrence, so the patient received six months of anti-tuberculosis treatment at a local hospital, which led to delay in diagnosis and treatment of the SqCLC. All these factors may explain the poorer outcomes in cavitated SqCLC cases. Incorporating local treatments, such as surgery, or combining EGFR TKIs with chemotherapy could potentially improve treatment outcomes. However, further studies are needed to confirm these approaches and their effectiveness.

Both cases in this study were treated with EGFR-TKIs as the first-line therapy rather than traditional chemotherapy. Case one showed a poor response to EGFR-TKI and the later surgery, adjuvant therapy of combined chemotherapy and immunotherapy seemed provide survival benefit (24). In Case two, the response to EGFR-TKI was a partial response for 14 months. Compared to case one, this suggests that non-cavitated lesions may be more sensitive to EGFR-TKI treatment. Both cases had an improved OS than those reported in previous literature (25), suggesting that the combination of chemotherapy and immunotherapy may be recommended for EGFR-TKI resistant driver gene-positive SqCLC.

The following limitations were encountered in the study. Only biopsy samples were tested in case two, making it difficult to confirm whether SqCLC was the only component in the tumor. Limited sample size justifies further studies with sufficient cases to validate these findings.

## Conclusion

In EGFR-mutant SqCLC, the cavitated subtype may exhibit reduced sensitivity to EGFR-TKIs. For patients without evident distant metastasis, early surgical intervention combined with chemotherapy and immunotherapy may represent a more effective treatment strategy.

## Data Availability

The original contributions presented in the study are included in the article/supplementary material. Further inquiries can be directed to the corresponding author.
